# Case Report of a Sharp-Pointed Foreign Body Ingested by a Child: How Long to Wait Before Surgical Intervention?

**DOI:** 10.7759/cureus.72780

**Published:** 2024-10-31

**Authors:** Anandit Bal, Santosh K Mahalik

**Affiliations:** 1 Pediatric Surgery, All India Institute of Medical Sciences, Bhubaneswar, Bhubaneswar, IND

**Keywords:** conservative and surgical treatment, foreign body ingestion complications, pediatric foreign body ingestion, sharp foreign bodies, treatment of foreign body ingestion, waiting period

## Abstract

Foreign body (FB) ingestion is one of the most common and challenging scenarios encountered in an Emergency Department. The incidence varies in different centers. They may be blunt objects, sharp-pointed objects, magnets, food boluses, and disk batteries. Most FBs pass spontaneously through the gastrointestinal (GI) tract, but the management of pointed, sharp FBs remains controversial. The relation between the length and width of sharp-pointed FBs and the mode of management remains questionable. In this case report, we discuss the management of a toddler who accidentally ingested an iron nail. We reviewed the existing literature and guidelines to find out the management of sharp-pointed foreign body ingestion in children.

## Introduction

Foreign body (FB) ingestion is one of the most common problems encountered by pediatric surgeons in emergency rooms and each one of them poses different challenges. The incidence of FB ingestion has increased in the past 13 years by 80% [1]. FBs can be broadly categorized into the following major groups: blunt objects, sharp-pointed objects, magnets, food boluses, and disk batteries [2]. The reported incidence of sharp FB varies between different centers. In European and Asian centers, the incidence rates were reported between 11% and 13% respectively [3]. In a recent report, among 3168 children with FB ingestion, approximately 18.3% were sharply pointed FBs. The management of sharp-pointed FB has been a matter of controversy as it has been associated with high morbidity and mortality. Delaying management could result in morbid complications including intestinal perforations [2,4]. Some guidelines suggest prompt removal of all sharp-pointed foreign bodies within reach of the endoscope and some others describe that if a foreign body has not progressed on imaging for 72 hours, surgical intervention may have to be planned [3]. This case report describes the management of a toddler who accidentally swallowed an iron nail. Additionally, we reviewed the literature and guidelines available on the same topic.

## Case presentation

**Figure 1:**
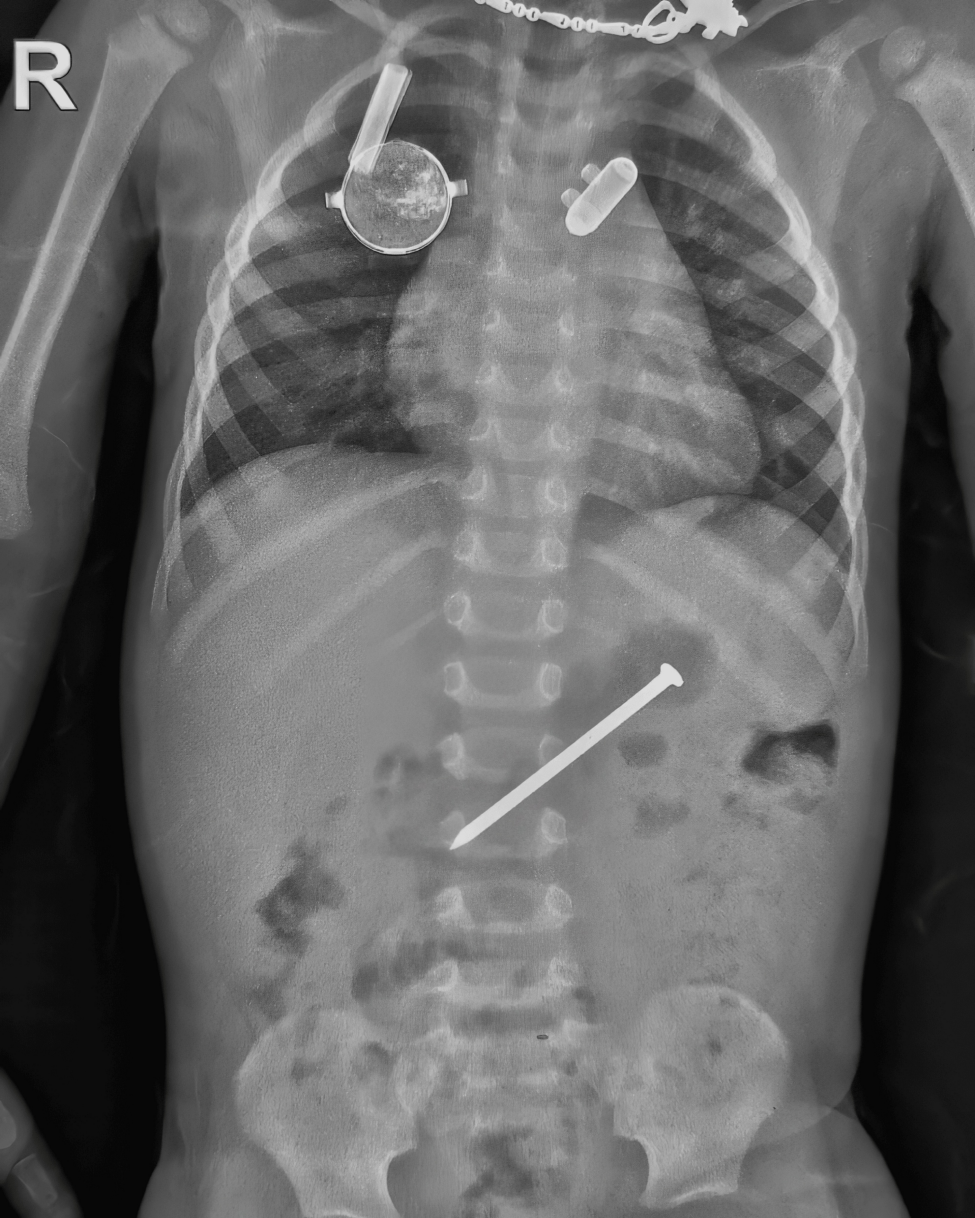
First X-ray of chest and abdomen, showing the sharp-pointed foreign body (FB) in the epigastric region

A two-year-old boy was admitted to the pediatric emergency with a history of accidental ingestion of a large construction iron nail for one day and there was a history of one episode of hematemesis before admission. At presentation, his vitals were stable, there was no respiratory distress or vomiting and he was feeding well. The x-ray was suggestive of the nail in the upper left quadrant of the abdomen (Figure 1).

**Figure 2:**
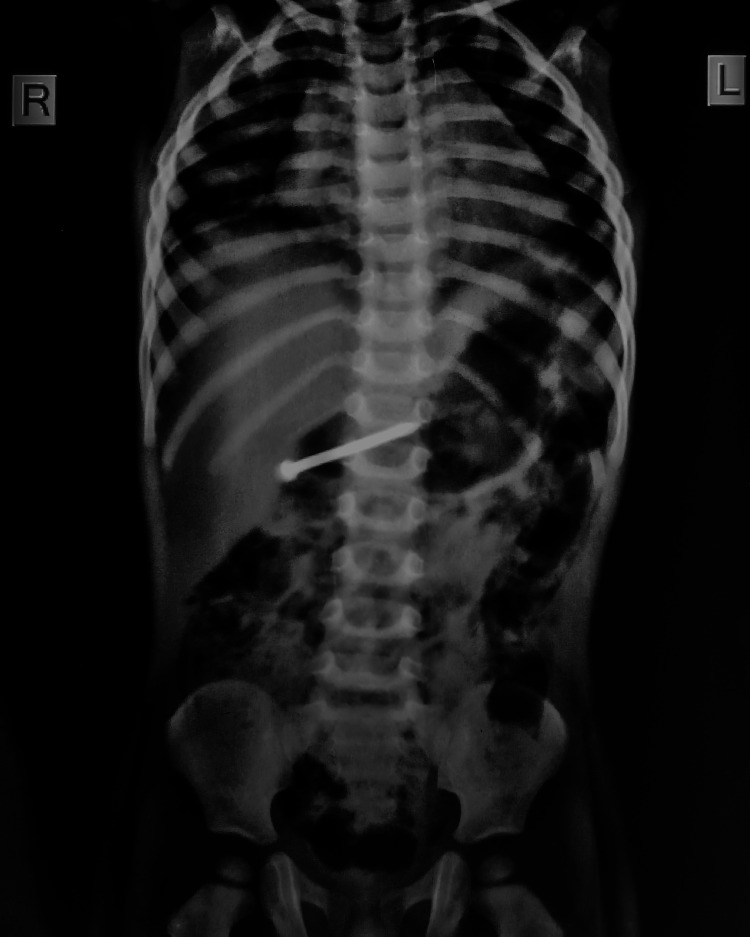
In the x-ray after 12 hours, the position of the foreign body (FB) has changed, most probably at the level of pylorus and duodenum

A repeat x-ray after 12 hours was suggestive of the nail in the same location with a changed direction (Figure 2).

**Figure 3:**
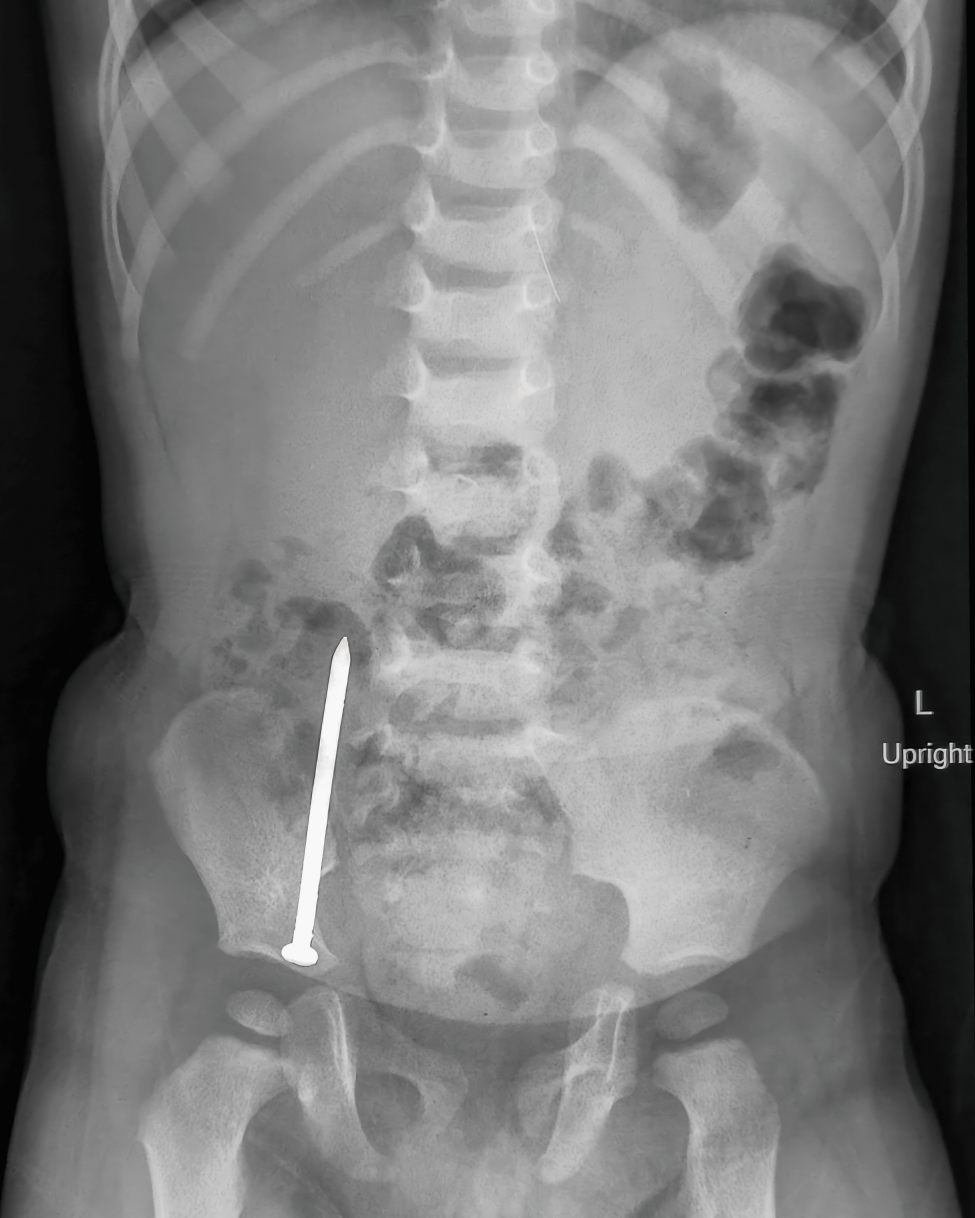
X-ray on day 6 of ingestion: the pointed foreign body (FB) is seen in the right iliac fossa with its point along the direction of the ascending colon

**Figure 4:**
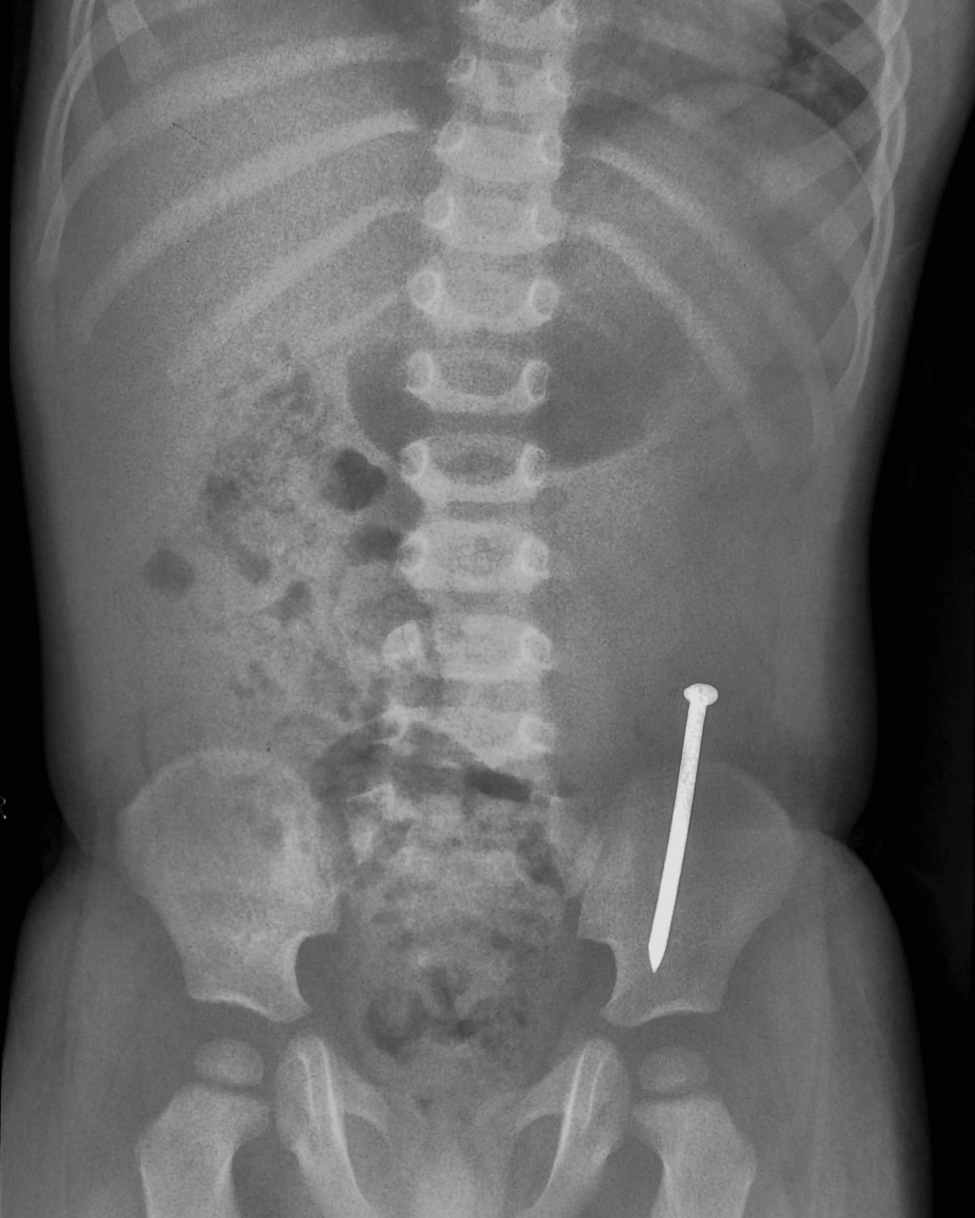
X-ray on day 8 of ingestion: the pointed foreign body (FB) is seen in the left iliac fossa with its point along the direction of the sigmoid colon towards the rectum

Since the child was asymptomatic, conservative management was planned for another 12 hours with close monitoring, but a repeat x-ray was again suggestive of the nail to be in the same position. The child then underwent upper gastrointestinal (GI) endoscopy by the Department of Gastroenterology but no FB was seen in the stomach, duodenum, or proximal jejunum. The child was then shifted to the pediatric surgery department for further management. As the child was asymptomatic, feeding well, and passing normal stool, we decided to keep the child under close observation with serial x-rays at 48-hour intervals (Figures 3, 4).

The plan of the management was discussed with the parents and they gave their consent to wait and watch.

**Figure 5:**
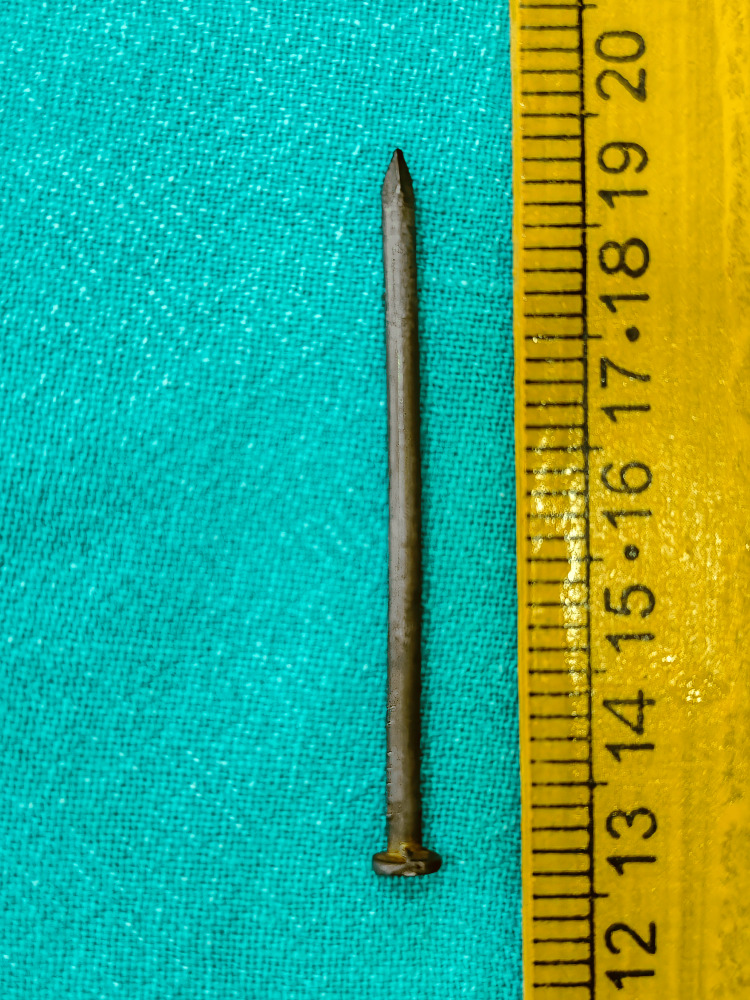
The spontaneously evacuated construction iron nail, 7 cm in length

The child eventually passed the nail after nine days without any complications. The nail measured approximately 7 cm in length (Figure 5).

On serial follow-up up to three months, the child was asymptomatic.

## Discussion

Foreign body (FB) ingestion is seen commonly in infants and toddlers attributed to their attitude to putting objects in their mouths. Plain radiographs are an inexpensive and easily available imaging modality for the diagnosis of radio-opaque FBs and give an idea about their location in the GI tract. Jackson’s axiom says “advancing points puncture, trailing do not”; this is true for many sharp foreign bodies in the GI tract [3]. According to previous reports, objects wider than 2 cm tend to get lodged in the stomach as they cannot get negotiated through the pylorus, and objects with a length of more than 5 cm tend to be held in the duodenum [5, 6]. The average transit time suggested for a foreign body in children is 3.6 days [7] and the average time from ingestion to perforation due to a sharp foreign body is estimated at 10.4 days [7, 8].

As per the North American Society for Pediatric Gastroenterology, Hepatology, and Nutrition (NASPGHAN) guidelines, endoscopic removal is recommended in all cases of esophageal FB. If the FB is in the stomach, endoscopic removal is recommended unless the object is shorter with a heavier blunt end. For sharp objects that are in the small intestine (beyond the ligament of Treitz) and the patient is asymptomatic, if the FB has shown no change in position in three days or if the patient becomes symptomatic, it necessitates enteroscopy or surgical intervention for the removal of the FB [3]. 

However, there is a lack of data regarding the rate of complications secondary to sharp-pointed FB ingestion and pediatric scientific literature is limited to a few case reports and case series, reporting variable complication rates [9, 10]. In a recent retrospective study by Quitadamo et al. [2], out of 580 children with sharp FB ingestion, only 3.8% were symptomatic (vomiting including hematemesis, drooling, bloody stool, abdominal pain, dysphagia, etc.). Endoscopic removal was considered for endoscopically reachable FBs (esophageal, gastric, proximal duodenal retention) while children with intestinal-retained sharp-pointed FBs were followed up until expulsion. All children with intestinal FB expelled the objects between eight and 94 hours without any complications. However, they did not record and analyse their data according to the size of the FBs.

Mantegazza et al. developed a nomogram based on the multivariable logistic regression model to predict the probability of the need for surgical or endoscopic intervention in children with FB ingestion, based on the information collected at admission [11]. The points include age, gender, FB type, pre-existing disease, signs and symptoms such as drooling of saliva, dysphagia, vomiting, retrosternal pain, abdominal pain, respiratory symptoms, hematemesis, food refusal, and unexplained crying [11]. Notably, not all symptoms carry the same weight in the scoring system. Symptoms related to an esophageal location contribute the highest number of points. Specifically, drooling, dysphagia, and retrosternal pain are associated with a 50%, 30%, and 30% increased risk of intervention, respectively, compared to patients who do not exhibit these symptoms. Other symptoms, such as vomiting, food refusal, unexplained crying, hematemesis, and abdominal pain, except for respiratory symptoms, contribute to a 10%-20% increased risk of intervention [11]. The type of FB is also given importance. While the blunt and sharp-pointed FB scored fewer points (<30), the magnet and button battery scored higher points (40-70).

In our case, the child should have undergone endoscopic removal of the FB at the time of presentation. However, as we received the child more than 48 hours after the ingestion with FB in the small intestine and the child was clinically asymptomatic, we opted for watchful expectant management in consultation with the parents. We could observe the change in the position of the FB in each x-ray and hence remained hopeful of spontaneous evacuation. No change in position on serial x-ray is ominous for impending complication [9, 10]. Even if we consider using the nomogram developed by Mantegazza et al. [11], as the child was asymptomatic and the sharp-pointed FB scores fewer points, it would not have recommended a surgical intervention. Hence, we believe in an asymptomatic child, regardless of the size of the sharp-pointed FB, if it is in the intestine and the position is changing on serial x-rays, we should wait for a spontaneous evacuation.

## Conclusions

In asymptomatic children with sharp-pointed foreign body ingestion, the size of the object may not be a determining factor in management. If it is located in the intestine, a trial of conservative management in the form of close clinical and serial radiological monitoring should be opted for. As long as the child is asymptomatic and the FB is changing position on serial x-rays, watchful expectancy is advocated even if it takes longer than usual time for spontaneous expulsion. There should be a very low threshold for surgical intervention if the child becomes symptomatic and if the FB does not change position.
